# Staged hybrid ablation in left atrial appendage aneurysm a rare cause of refractory atrial tachyarrhythmia—a case report

**DOI:** 10.1093/ehjcr/ytae298

**Published:** 2024-06-14

**Authors:** Ashish Mittal, Manoraj Navaratnarajah, Stephen Harden, Theodore Velissaris, Paul R Roberts

**Affiliations:** Dept. of Cardiac Electrophysiology, St Bartholomew’s Hospital Heart Centre, London EC1A 7BE, UK; Dept. of Cardiothoracic Surgery, University Hospital Southampton, Southampton SO16 6YD, UK; Dept. of Cardiothoracic Radiology, University Hospital Southampton, Southampton SO16 6YD, UK; Dept. of Cardiothoracic Surgery, University Hospital Southampton, Southampton SO16 6YD, UK; Dept. of Cardiovascular Medicine, University Hospital Southampton, Southampton SO16 6YD, UK

**Keywords:** Left atrial appendage aneurysm, Case report, Refractory atrial tachyarrhythmias, Cardiac magnetic resonance, Cardiac computed tomography

## Abstract

**Background:**

Left atrial appendage aneurysm (LAAA) is a rare cardiac anomaly, which can be congenital or acquired in origin. Because most cases are asymptomatic, it is typically diagnosed incidentally in the second to third decades of life. We present a case of a 28-year-old male with refractory atrial tachyarrhythmias and significantly reduced exercise tolerance. The informed consent was given by patient for this manuscript.

**Case summary:**

We present a case of a 28-year-old male with refractory atrial tachyarrhythmias and significantly reduced exercise tolerance after an episode of COVID respiratory infection. He was referred by primary care physician for management of atrial fibrillation (AF) with CHA2DS2Vasc score zero. He had documented AF and atrial flutter (AFL) resistant to both chemical and electrical cardioversions. Initial portable focused transthoracic echocardiography documented borderline reduced left ventricular ejection fraction in context of AFL. Electrophysiological study confirmed the diagnosis of typical AFL. Successful radiofrequency ablation of cavo-tricuspid isthmus resulted in bidirectional isthmus conduction block. However, patient developed AF, which was electrically cardioverted at the end of procedure. Patient was discharged on bisoprolol, ramipril, and apixaban, and outpatient cardiac MRI was organized to look for post-COVID myocardial scarring. Patient had recurrence of symptoms, and this time it was due to AF. Multimodal imaging led to discovery of LAAA, in which after discussion in multidisciplinary meeting, he was accepted for and managed with surgical resection of LAAA with concomitant Cox-Maze IV procedure. On 9 months post-operative follow up, patient is maintaining sinus rhythm and has completely returned to baseline activities.

**Discussion:**

A young patient with refractory atrial arrhythmia should be referred for multimodal cardiovascular imaging to rule out any structural heart disease. Left atrial appendage aneurysm is rare and can be managed conservatively, but surgical excision is most reported and appears to favour arrhythmia-free survival.

**Learning points**

- Left atrial appendage aneurysm is a rare malformation, which may be responsible for refractory atrial tachyarrhythmias, chest pain, heart failure, or systemic thromboembolism.
- A young patient with refractory atrial arrhythmia should be referred for multimodal cardiovascular imaging to rule out structural heart disease.
- Although conservative management is an option, surgical excision is most reported and appears to favour arrhythmia-free survival.

## Introduction

Left atrial appendage aneurysm (LAAA) is a rare cardiac anomaly, which can be congenital (90%) or acquired in origin.^1,2^ Since first described in 1960, approximately 150 cases have been reported so far.^1^ Congenital cases are most likely due to dysplasia of atrial pectinate muscles, and acquired cases are associated with organic mitral valve disease and conditions with raised left atrial (LA) pressures. Most cases are asymptomatic, and it is typically diagnosed incidentally in the second to third decades of life. Symptoms are mainly comprising of palpitations (44.6%), dyspnoea on exertion (28.7%), and chest pain (11.9%) and systemic thromboembolic events (5.95%). Among patients with palpitations, ECG was found to be in atrial fibrillation (AF; 26.7%), in supraventricular tachycardias (9.9%), and in sinus tachycardia (59.4%).^3^ According to the literature, LAAA may result in compression of left anterior descending artery or its derivatives resulting in chest pain and increased tension on or congenital malformation of conduction system causing atrial arrhythmias (AA). Majority of cases are managed with surgical resection of LAAA. Common histological findings include fibrosis of endocardium or myocardium and fatty infiltration. We present a case of a 28-year-old male with refractory atrial tachyarrhythmias and significantly reduced exercise tolerance. The informed consent was given by patient for this manuscript.

## Summary figure

**Figure:**
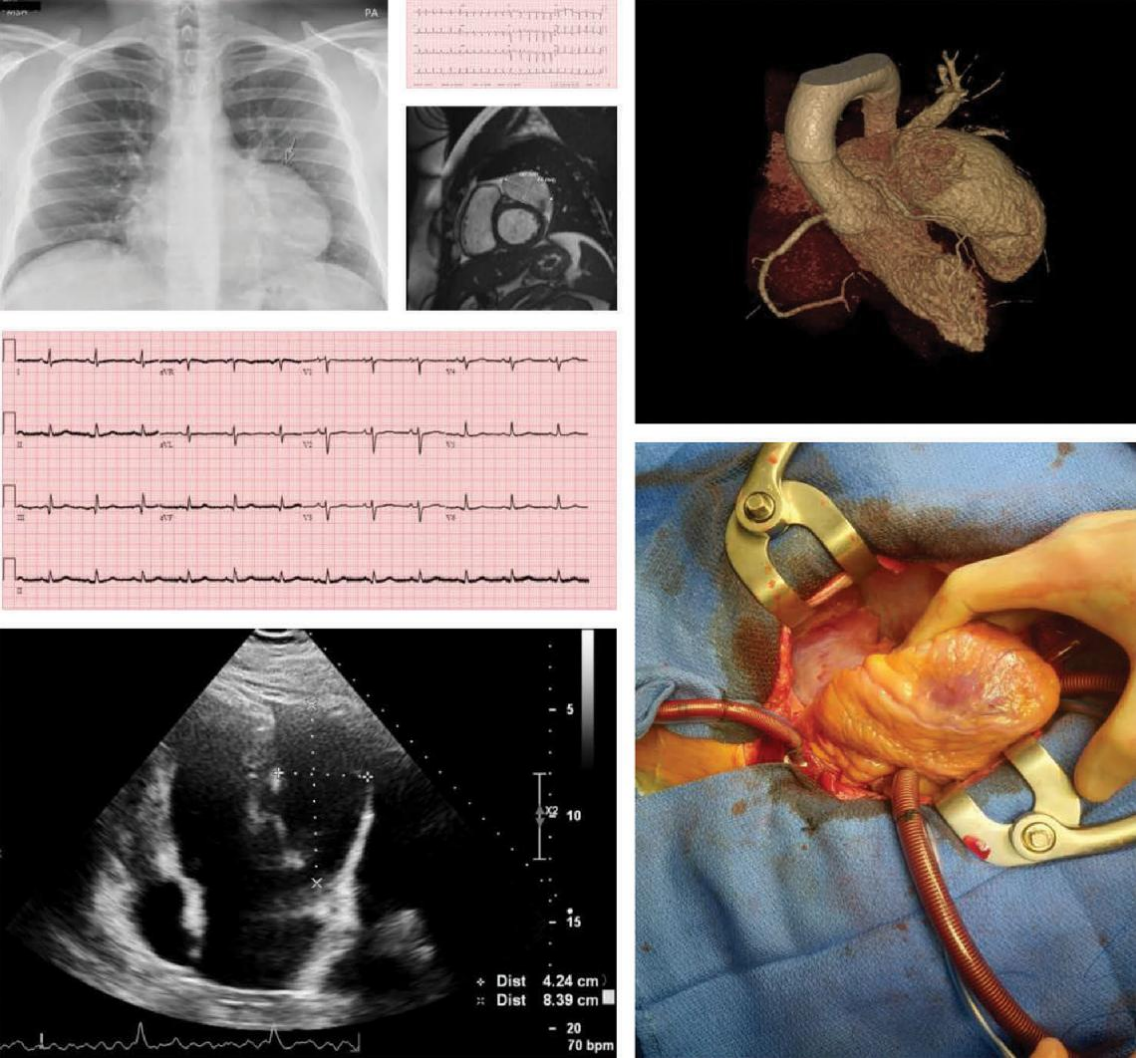


## Case presentation

**Figure 1:**
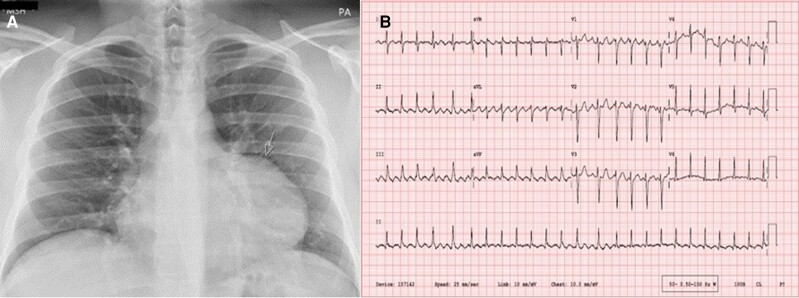
(*A*) Posterior–anterior chest X-ray showing a prominent bulge in the left side of the cardiac silhouette (arrow). (*B*) A 12-lead ECG in ED with narrow complex tachycardia suggestive of typical AFL with ventricular rate of 150 b.p.m.

In September 2021, a 28-year-old male was referred by primary care physician with a new diagnosis of AF with palpitations, reduced exercise tolerance, and pre-syncope that had started after COVID respiratory infection in February 2021. He subsequently presented to the emergency department (ED) with atrial flutter (AFL) in November 2021. The patient’s CHA2DS2Vasc score was zero. He performed regular gym exercises although of not to an endurance level, of normal body weight, and reported no excess intake of alcohol. On physical examination, his blood pressure was 110/76 mmHg and heart rate (HR) 150 b.p.m., with no signs of left heart failure. A 12-lead ECG documented AFL with variable atrio-ventricular conduction, and chest X-ray showed a distortion to the contour of the left cardiac border (*Figure 1*). However, bedside transthoracic echocardiography (TTE) in ED reported borderline reduced left ventricular ejection fraction (LVEF) with all chambers normal in dimensions and no evidence of valvular pathology. After medical treatment with bisoprolol 5 mg/day, ramipril 2.5 mg/day, and apixaban 5 mg twice a day, an outpatient electrical cardioversion was organized in 6 weeks’ time. In December 2021, post cardioversion, patient felt clinically well. His symptoms recurred with exercise 2 months after cardioversion. Holter monitor confirmed the presence of AFL. We presumed AFL was a trigger for AF; hence, we offered him interventional treatment with radiofrequency ablation (RFA) for AFL. In August 2022, electrophysiological study confirmed diagnosis of typical AFL, and successful RFA of cavo-tricuspid isthmus resulted in bidirectional isthmus conduction block. However, patient developed AF, which was electrically cardioverted at the end of procedure. Patient was discharged on apixaban 5 mg twice a day, bisoprolol 5 mg/day, and ramipril 2.5 mg/day, and a structural cardiac magnetic resonance scan (CMR) was organized to look for post-COVID myocardial scarring. Patient had recurrence of symptoms, and this time it was due to AF.

**Figure 2:**
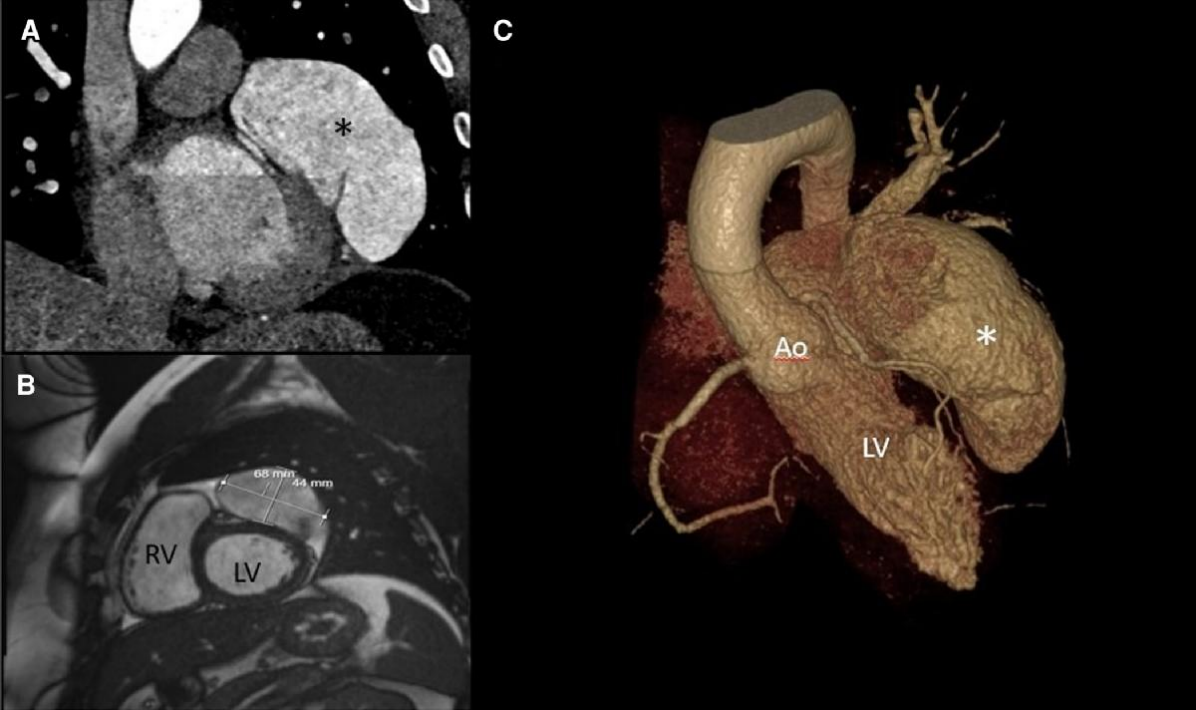
(*A*) CT scan of the heart in coronal plane depicting the LAAA (asterisk) in contact with the LV lateral wall. (*B*) CMR steady-state free precession image in diastole showing the LAAA exerting compression on the anterior and anterolateral LV walls. (*C*) CT 3D reconstruction showing the LAAA (white asterisk) in close approximation to the LV myocardium, the left anterior descending artery, and left superior pulmonary vein.

In October 2022, CMR demonstrated a LAAA measuring 6.8 × 4.4 cm in dimensions with external compression of the basal to mid-anterior and anterolateral LV wall. Left ventricular systolic function was mildly impaired (LVEF 49%) with no cardiac chamber dilatation. There was some mild non-specific increased signal on late gadolinium enhancement imaging in the basal lateral wall, but no myocardial oedema was identified. In November 2022, computed tomography coronary angiogram (CTCA) confirmed the diagnosis of LAAA, with no associated thrombus, and ruled out coronary artery disease (*Figure 2*; CMR and CT images). The patient was referred for urgent surgical resection of LAAA while maintained on medical therapy after discussion in Heart Team multidisciplinary meeting in January 2023.

**Figure 3:**
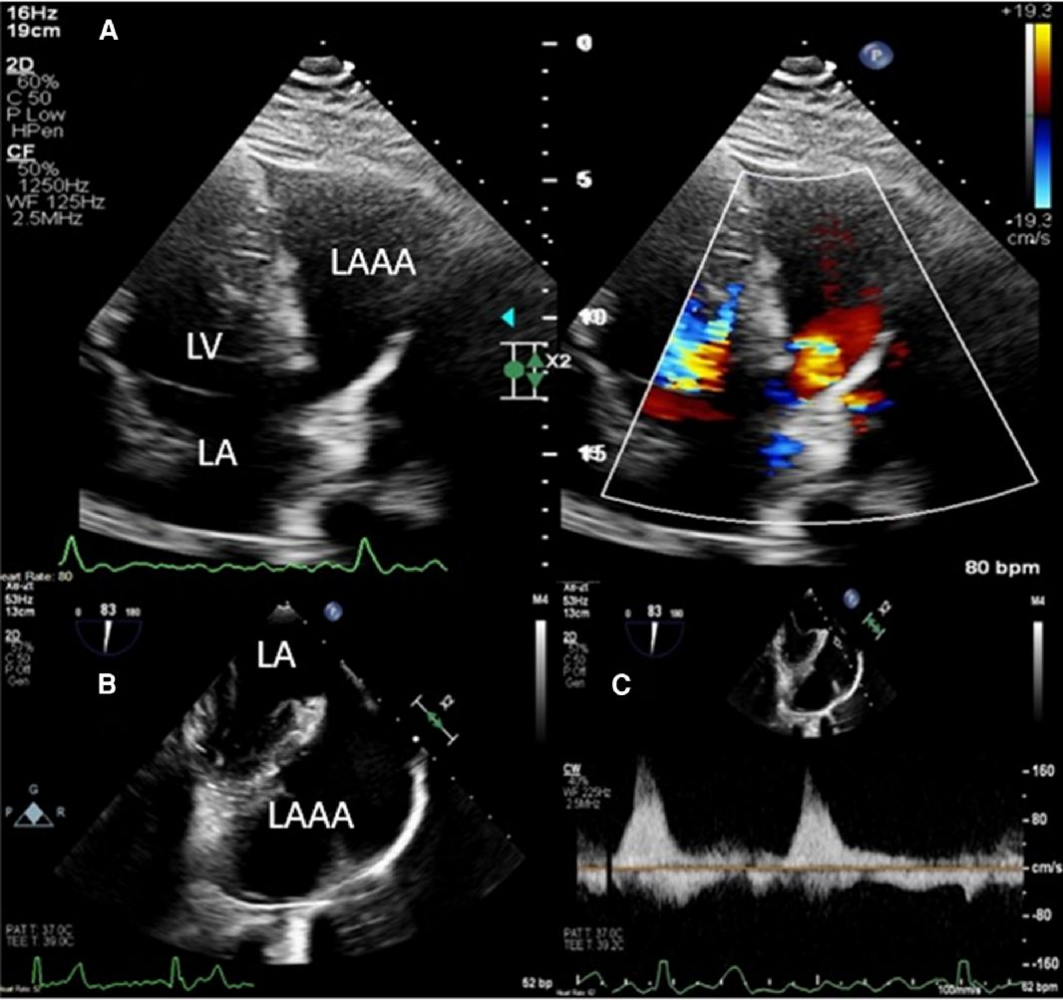
(*A*) Transthoracic echocardiography during pre-operative assessment in slight off-axis apical four-chamber view showing round hyperlucent structure on the lateral side of LV, which is in continuity with LA as documented with colour Doppler signals. (*B*) Intraoperative TOE at mid-oesophageal level showing LAAA with no evidence of intracavitary thrombus or spontaneous echo contrast. (*C*) Continuous wave Doppler at the level of LAAA inlet documenting normal emptying velocity of 160 cm/s.

**Figure 4:**
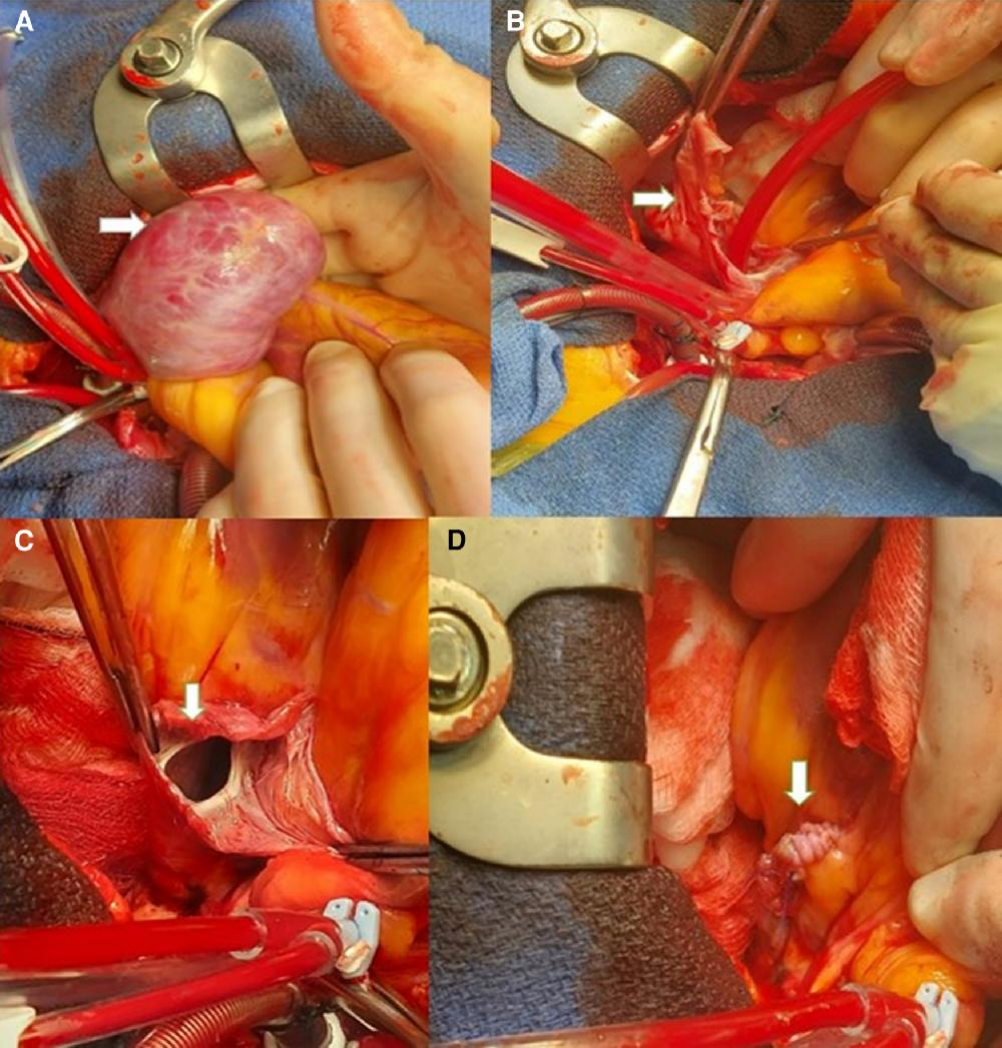
Intraoperative images of resection of LAAA (arrows). (*A*) Large LAAA exposed. (*B*) Incision of LAAA. (*C*) Incised LAAA with open communication to LA. (*D*) Oversewn base of LAAA post excision.

**Figure 5:**
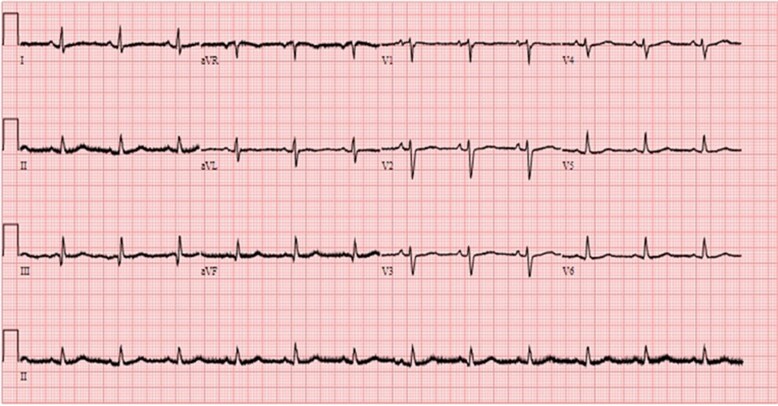
A 12-lead ECG, 8 weeks post-operative follow-up documenting normal sinus rhythm.

In March 2023, preoperative assessment with departmental TTE documented LAAA anterolateral to LV measuring 8.4 × 5.5 cm in dimensions with ostial diameter of 1.6 cm at the left atrium. Intra-operative transoesophageal echocardiography (TOE) confirmed LAAA with no thrombus and normal emptying velocities (*Figure 3*). Surgery was undertaken via median sternotomy, and LAAA was excised and orifice was oversewn with continuous non-dissolvable suture in two layers. Concomitant Cox-Maze IV procedure with bipolar RFA ablation clamp (Isolator Synergy Ablation System by AtriCure Inc, Mason, OH, USA) and cryoablation in coronary sinus area was performed (*Figure 4*). The post-operative course was uneventful, and patient was discharged on oral amiodarone 200 mg/day, ramipril 2.5 mg/day, and apixaban 5 mg twice a day. In May 2023, 8 weeks post-operative follow-up, the patient had returned to normal physical activity, and LVEF was normalized to 60% with no evidence of recurrence of AA or symptoms; hence, amiodarone, ramipril, and apixaban were stopped (*Figure 5*). This has been maintained through to 9 months post-operatively.

## Discussion

This case highlights clinical challenges in the diagnosis and management of LAAA, a rare anomaly. During patient’s initial presentation to ED, his chest X-ray documented distorted left-side cardiac contour, which is a non-specific finding with possible differential diagnosis of pericardial cyst, mediastinal mass, cardiac aneurysm, or cardiac tumour. Initial bedside focused TTE in ED missed the finding of LAAA and reported marginally low LVEF with mild LA dilation. After appropriate HR control and oral anticoagulation, patient opted to have elective electrical cardioversion in 4–6 weeks’ time, instead of TOE-guided inpatient electrical cardioversion. We believe further imaging at this stage with CT thorax and/or TOE-guided cardioversion may have resulted in early diagnosis and management of LAAA. In a case series review, chest X-rays were found to be abnormal in all cases, and TTE although considered as primary investigation lacked sensitivity compared with TOE.^3^ Adding echo contrast and three-dimensional (3D) imaging to TTE has potential to improve diagnostic sensitivity and rule out intracavity thrombus prior to surgical or non-surgical intervention as demonstrated in a case report by Harland *et al*.^4^ Hence, TOE should be mandatory when TTE is ambiguous.

CMR and cardiac CT provide 3D anatomical assessment with high spatial resolution. Our patient had CMR to assess myocardial scar burden post-COVID pneumonitis, which could explain refractory AA, but discovery of LAAA was incidental. It is difficult to ascertain whether COVID pneumonitis or LAAA triggered AF in our case, but long-haul COVID virus presence in myocardium after lung infection has been shown to provoke AF long after recovery from pneumonitis.^5^

Early surgical excision is recommended even in asymptomatic cases to prevent serious complications such as AA, systemic thromboembolic events, myocardial ischaemia, and dysfunction associated with large LAAA. Due to lack of high-quality data, a multidisciplinary approach and a shared decision-making with the patient should be employed. The most common surgical technique involves median sternotomy although minimal endoscopic resection has been reported as well with good outcomes.^6^ Data are lacking in direct comparison of surgical intervention vs. conservative treatment. If a conservative route is chosen, the patient should be closely monitored and anticoagulated.^7^ Plonska *et al.* reported a case of LAAA who had been followed up for more than 20 years with no complications.^8^ Although LAAA excision results in complete resolution of AA, concomitant Cox-Maze IV is recommended in cases of dilated LA and chronic AF. However, this may increase the risk of requiring permanent pacemaker.^9^

Another important finding in our case is the presence of both AF and AFL. After AFL ablation, CMR discovered LAAA, and we believe AFL and AF were interlinked, but whether LAAA was directly responsible for both rhythms is difficult to establish. In a meta-analysis, it was found that the incidence of AF after successful AFL ablation with confirmed bidirectional block was high and recommended to perform concomitant AF ablation in cases where AF was documented prior to AFL ablation.^10^ There are many risk factors associated with AF, but we believe LAAA was responsible for refractory atrial tachyarrhythmias in our patient as he is still maintaining NSR 1-year post-surgical excision of LAAA.

## Conclusion

LAAA is a rare condition, which requires multimodal imaging to confirm the diagnosis and plan adequate treatment strategy, which in most reported cases is surgical excision to avoid serious complications.

## Lead author biography


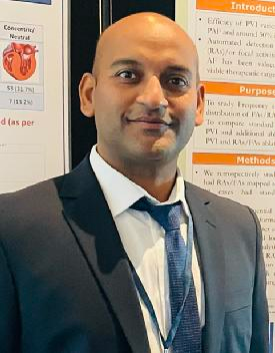


Dr Ashish Mittal, MD, studied medicine at the University of Medicine and Pharmacy ‘Victor Babes’ Timisoara, Romania, and completed training in general cardiology from the Institute of Cardiovascular Diseases Timisoara. He is currently training in advance interventional cardiac electrophysiology and device implantation at Barts Heart Centre, St Bartholomew’s Hospital, London, UK, where he is also participating in departmental audits and research.

##  

**Consent:** ‘The authors confirm that written consent for submission and publication of this case report including the images and associated text have been obtained from patient in line with COPE guidance’ this statement has been added as advised by CARE reviewer.

**Funding:** None declared.

## Data Availability

The data underlying this article are available in article and in its online Supplementary material.
